# AerialWaste dataset for landfill discovery in aerial and satellite images

**DOI:** 10.1038/s41597-023-01976-9

**Published:** 2023-01-31

**Authors:** Rocio Nahime Torres, Piero Fraternali

**Affiliations:** Politecnico di Milano, Department of Electronics Information and Bioengineering, Milan, 20133 Italy

**Keywords:** Research data, Environmental impact

## Abstract

Illegal landfills are sites where garbage is dumped violating waste management laws. Aerial images enable the use of photo interpretation for territory scanning and landfill detection but this practice is hindered by the manual nature of this task which also requires expert knowledge. Deep Learning methods can help capture the analysts’ expertise and build automated landfill discovery tools. However, this goal requires public high-quality datasets for model training and testing. At present no such datasets exist and this gap penalizes the research toward scalable and accurate landfill discovery methods. We present a dataset for landfill detection featuring airborne, WorldView-3, and GoogleEarth images annotated by professional photo interpreters. It comprises 3,478 positive and 6,956 negative examples. Most positive instances are characterized by metadata: the type of waste, its storage mode, the type of the site, and the evidence and severity of the illicit. The dataset has been technically validated by building an accurate landfill detector and is accompanied by a visualization and annotation tool.

## Abstract (summary)

**Table:** 

Measurement(s)	Landfills detections in very high-resolution remote sensing imagery
Technology Type(s)	remote sensing imagery
Sample Characteristic - Environment	landfill
Sample Characteristic - Location	Region of Lombardy

## Background & Summary

Finding illegal landfills is a prominent objective of environmental agencies but is a very complex and time-consuming task that requires sophisticated investigations. The increasing availability of aerial images has boosted the adoption of photo interpretation for territory scanning and suspicious site identification^1^. However, large scale territory analysis is hindered by the manual nature of the photo interpretation task, which must be conducted by experts. The advances in Computer Vision (CV) boosted by Deep Learning (DL) hold the promise to capture the expertise of professional photo interpreters and distill it into computer-aided tools supporting territory monitoring at scale. The precondition for reaping the benefits of CV for landfill discovery is the availability of high-quality datasets for training, validating, and testing predictive models. At present no such datasets exist for the landfill discovery task and this gap penalizes the research towards scalable and accurate detection methods^2–6^.

DL-based waste discovery in aerial images has been addressed by very few works^7–9^. These contributions illustrate the potential of DL for realizing image interpretation pipelines at different scales and based on different CV tasks, such as scene classification and object detection. However, so far none of the related works has publicly released the data set and the ground truth annotations employed for building the described architectures. The main reason for this restriction is the sensitivity of the data due to their potential connection with investigation activities. General purpose datasets for aerial image/scene classification and object detection cannot help either. The most similar category found in public datasets is that of *dump sites* in the BigEarthNet repository^10^, which represents rather generic scenes and comprises only very small and coarse images. This limits the replicability of research and ultimately the progress of the state of the art.

The contribution of this work is the AerialWaste data set, a professionally curated dataset for landfills discovery in aerial images, constructed based on the following criteria:

- The images have different provenance and quality.
- Images are associated with annotations that specify the presence of a waste dump as a whole and the visibility of specific types of garbage.
- The annotations are curated by professional photo interpreters specialized in the use of aerial images for landfill detection.
- The provided annotations can be used as ground truth (GT) labels for multiple CV tasks: binary and multi-class image classification and weakly supervised localization.
- The dataset adheres to the standard MS COCO format^11^.

AerialWaste contains 10,434 images generated from tiles of three different sources: AGEA Ortophotos (≈20 cm GSD), WorldView-3 (≈30 cm GSD) and GoogleEarth (≈50 cm GSD). Of these images 3,478 are positive examples representing locations considered suspicious and 6,956 are negative examples. Most positive instances are characterized by metadata about the evidence, severity, and area type of the site. A subset of 715 samples is annotated with the class of the waste objects visible in the image chosen among 22 different categories. 169 images are provided with segmentation masks (from which bounding boxes are automatically extracted) that surround the objects of 9 garbage classes.

The data set and its documentation can be accessed from the website www.aerialwaste.org.

## Methods

**Fig. 1:**
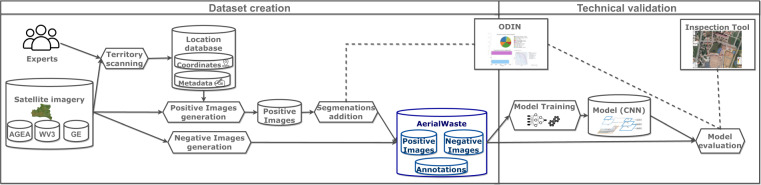
The phases of the AerialWaste creation and technical validation process. In the data set creation phase, experts scan the aerial images of a region and create a database of identified locations with associated metadata (waste type, area type, storage mode, evidence, and severity). The location database is used to extract the positive images from the three data sources. The negative images are sampled from the same sources at random locations. A subset of the positive images is enriched with segmentation masks with the help of the ODIN annotator tool. In the technical validation phase, the AerialWaste data set is used to train and test a CNN binary scene classification model. A quantitative evaluation is performed by assessing the prediction performance of the CNN model with the standard metrics of precision, recall, and F1 score. A qualitative evaluation can be also done by visualizing the data set and the predictions with the help of the Inspection Tool.

AerialWaste is the result of a collaboration with the professional photo interpreters and territory monitoring experts of ARPA Lombardia, the environment monitoring agency of Region Lombardy (Italy). Figure 1 illustrates the creation and technical validation process of the AerialWaste dataset.

The dataset creation phase exploited the experts’ knowledge in order to produce a collection of images divided into positive and negative samples and enriched with meta-data and segmentation masks. The professional photo interpreters scanned a geographic region and built a database of relevant locations where waste dumps were visible. From such a database the actual image collection was created. Samples corresponding to the selected locations were extracted from the three above-mentioned aerial image sources and tagged as positive. The data set was completed with negative samples extracted from the same sources at randomly chosen locations. The positive sites were annotated with meta-data defined by the experts, including the type of waste visible, its storage mode, the type of the area around the site, the degree of evidence, and the severity of the illicit.

The technical validation phase was conducted by using the AerialWaste images and annotations to train, validate and test a state-of-the-art binary scene classification model for detecting the presence of waste. The model trained on images from all the three heterogeneous sources achieves 87.9% average precision on the test set. The model trained only on high-quality orthophotos attains 94.5% average precision on the test set. These results show the effectiveness of the AerialWaste data set for training waste discovery models.

### Data set creation

**Fig. 2:**
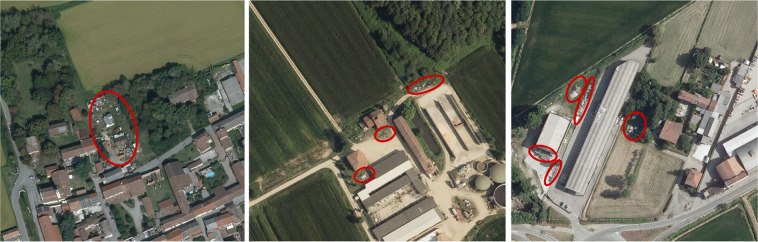
Examples of the presence of waste in potentially illegal sites. Red circles indicate suspicious objects. In all the images accumulations of various materials and scattered waste are present.

AerialWaste copes with the visual heterogeneity of the scenes in which waste dumps occur in aerial images and with the diverse nature of the objects that compose a waste deposit. When observed from above waste dumps appear as complex arrangements of objects of different shapes, sizes, and orientations: a typical case occurs when a shed or a dismissed industrial plant is filled with waste which can be seen spilling over the construction boundaries. The area may also contain other clues, such as sparse debris, pallets, or containers. Further signs can be trucks, the isolation of the place, secondary access roads, and stressed vegetation^12^. Typical materials found in dumping sites include organic waste, plastics, glass, metal, paper, wood, textiles, tires, bulky waste, electronics, and asbestos panels^13^. Figure 2 presents some examples of how waste dumps appear in aerial images.

#### The location database

The creation of the dataset starts with the definition of a database of locations identified as containing waste dumps by four territory monitoring professionals of the Environmental Protection Agency of Region of Lombardy (ARPA). The database comprises 10,434 locations belonging to 487 different municipalities of the Lombardy region in Italy. Of these 33% are sites reported by the analysts as positive, i.e., hosting some form of illegal waste disposal. The remaining 67% are randomly sampled in the territory of the same municipalities. To account for the class imbalance, the negative locations are twice as many as the positive ones. The negative instances are obtained by randomly picking two locations at a distance *d* from each positive instance (with 1 km ≤ *d* ≤ 5 km). The lower threshold of 1 km is selected to avoid including a positive site in a negative image, whereas the upper threshold of 5 km is selected heuristically so that the negative sample is likely to remain in an area with the same land use as the positive one. All data points are characterized by the geographic coordinates (latitude and longitude). Most sites have additional metadata obtained from the ARPA database: the evidence (how many indicators of non-compliance with waste disposal regulations are detected by the analyst) and severity (how critical the non-compliance is perceived by the analyst) expressed in the 0 (low) to 3 (high) range, the type of visible objects (e.g., tires, scrap), the storage mode (e.g., delimited heaps, containers) and the category of the area (e.g., production plant, agricultural area).

The location database is used to generate the actual image dataset and is not disclosed for confidentiality reasons.

#### Images generation

For each location in the database one square image is created centered at that location and covering an area of 210 × 210 meters. The images are drawn from tiles of three provenance sources:

- AGEA: orthophotos generated by an airborne campaign executed by the Italian Agriculture Development Agency AGEA. This source contains the images produced by the acquisition campaign launched in 2018 in the Lombardy Region (Italy). An RGB aerophotogrammetry survey was conducted at a spatial resolution of ≈20 cm GSD. The size of the resulting images is ≈1000 × 1000 pixels.
- WorldView-3 (WV3): high-resolution pan-sharpened RGB images acquired by a commercial satellite sensor (no pan-sharpened near infrared images were used). The collection corresponds to the campaign conducted in 2021 at a spatial resolution of ≈30 cm GSD. The size of the resulting images is ≈700 × 700 pixels.
- Google Earth (GE): images downloaded using the Google API. Spatial resolution is ≈50 cm GSD. The size of the images is ≈1000 × 1000 pixels as a result of the up-sampling by a scale of 2 performed by the API. Google images are free to the public and have been used in different remote sensing studies^14–18^. Their use must respect the Google Earth terms and conditions^19^. The API provides only the most recent imagery and thus small changes can be observed when an image is updated after the photo interpreter has annotated it.

**Fig. 3:**
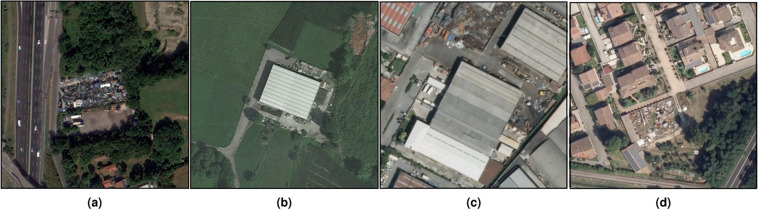
Examples of images associated with positive locations in the database. From left to right: (**a**) large quantities of metal scraps deposited in an unauthorized area (source: WV3); (**b**) production plant with heaps of waste, excavated earth, and rocks near the border of the property (source: GE); (**c**) mixed scrap and bulky items with volume excessive w.r.t. the site dimension (source: AGEA); (**d**) heterogeneous materials stacked in a closed non-productive residential zone (source: AGEA).

Figure 3 shows some examples of positive locations from different sources. The images present a high intra-class diversity: (1) the type of area includes isolated sites, urban locations and industrial areas; (2) the visible waste objects are extremely heterogeneous; (3) the spatial arrangement and the scale of the waste dumps in the scenes are diverse.

**Fig. 4:**
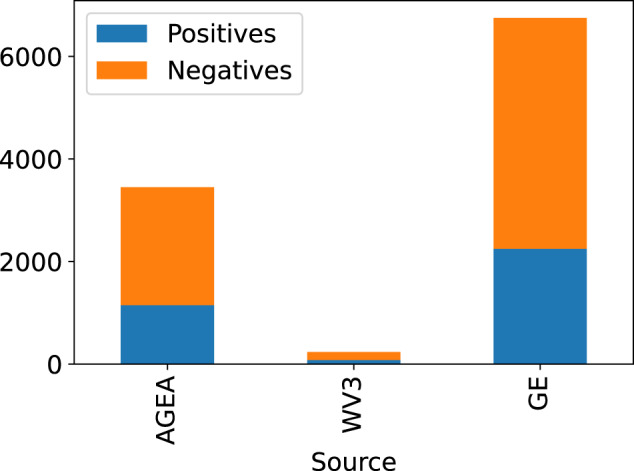
Distribution of the positive and negative samples by provenance source.

Figure 4 shows the distribution of positive and negative images over the provenance sources. Most examples are drawn from Google Earth, followed by AGEA orthophotos. Fewer samples are drawn from the WorldView-3 collection because these were acquired only recently (in 2021).

**Fig. 5:**
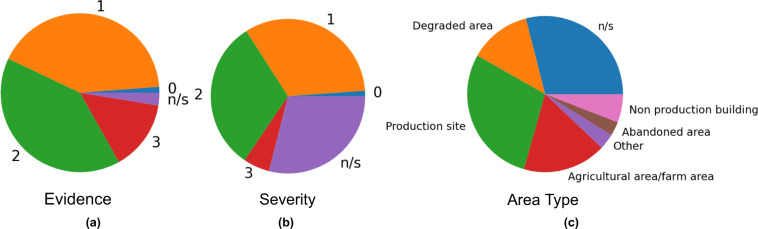
Distribution of images in the dataset by the (**a**) evidence, (**b**) severity and (**c**) area type properties (n/s = not specified).

Figure 5 shows the distribution of the evidence, severity, and area type properties across the positive samples. These characteristics are not used for training the CV component but are mentioned to show the heterogeneity of the dataset, which contributes to the complexity of the waste detection task. The evidence attribute is present in most samples, and the severity and area type annotations are specified for ≈72% of the samples.

### Meta-data definition and data set annotation

The type of waste and its visual appearance are expressed by means of two annotations: the type of visible objects (TO) and the storage mode (SM). Table 1 lists the different TO and SM labels used by the experts and reports the number of samples annotated with them. Some of the categories used by the experts in their current interpretation activity are equivalent to the standard codes proposed by the European Union (e.g., “07.5-Wood wastes” or “07.4-Plastic wastes”)^20^. The current categories have been chosen by the photo interpreters as those most clearly distinguishable in the aerial images. The expansion of the data set annotations to comply with the standard EU codes is planned as a future activity.

**Table 1:** Type of Object (TO) and Storage Mode (SM) labels and total amount of samples per label.

Label type	Label	Total
TO	Rubble/excavated earth and rocks	294
TO	Bulky items	286
TO	Fire wood	173
TO	Scrap	167
TO	Plastic	126
TO	Vehicles	53
TO	Tires	45
TO	Domestic appliances	24
TO	Paper	26
TO	Sludge-Zootechnical waste-Manure	19
TO	Stone/marble processing waste	13
TO	Asphalt milling	12
TO	Corrugated sheets (presumed asbestos-cement)	11
TO	Glass	8
TO	Foundry waste	9
SM	Heaps not delimited	448
SM	Containers	167
SM	Big bags	50
SM	Pallets	50
SM	Delimited heaps (by barriers/walls/etc)	69
SM	Cisterns	35
SM	Drums bins	18
TOTAL		2103

**Fig. 6:**
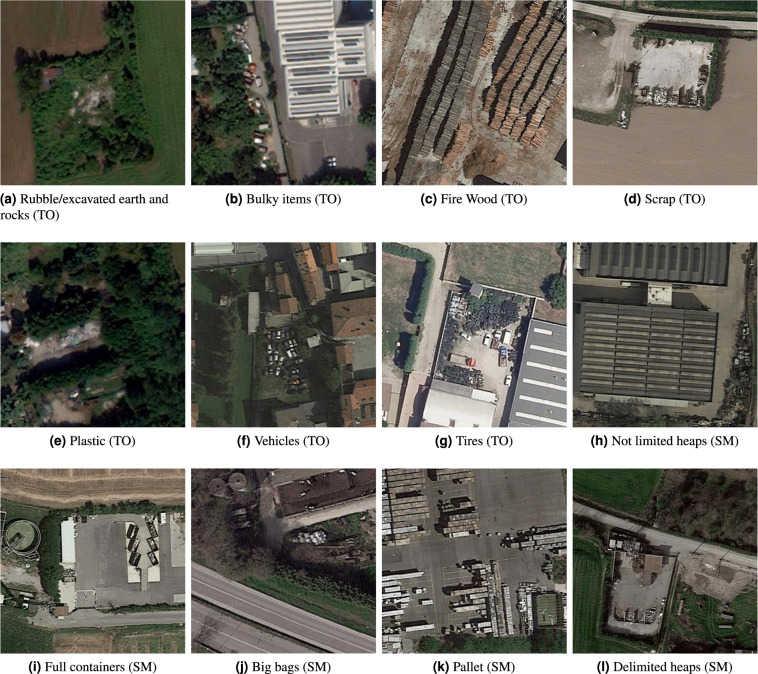
Examples of images of sites annotated with different labels.

Figure 6 presents examples of images annotated with different TO and SM labels. While some objects such as bulky items, excavated earth, tires, and vehicles are easy to recognize, some others such as scrap or plastics require a more trained eye. The storage modes are more distinguishable.

**Fig. 7:**
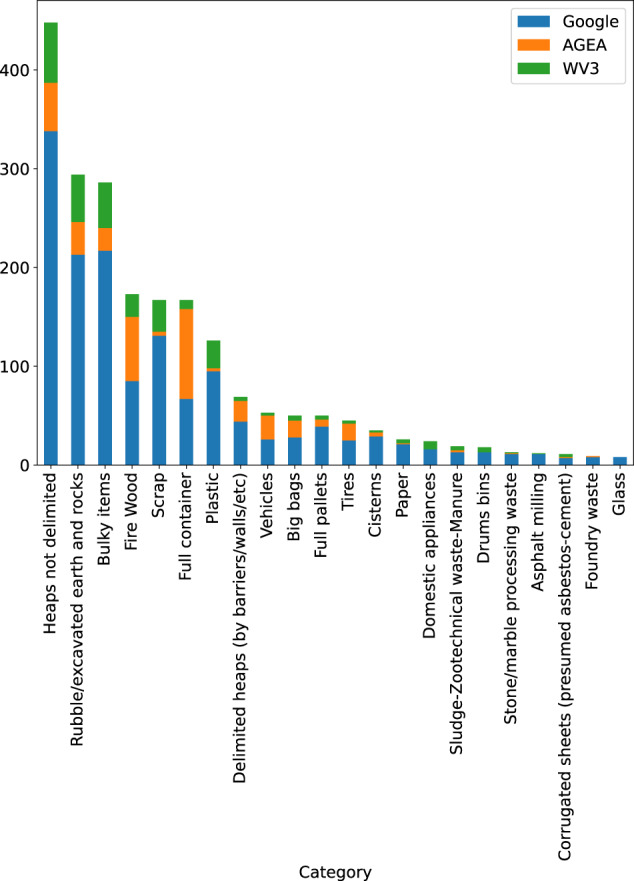
Distribution of the images across the TO and SM classes and with respect to the provenance sources.

Figure 7 shows the distribution of the images across the TO and SM classes and with respect to the provenance sources. Most annotations are associated with images drawn from the Google Earth source.

**Fig. 8:**
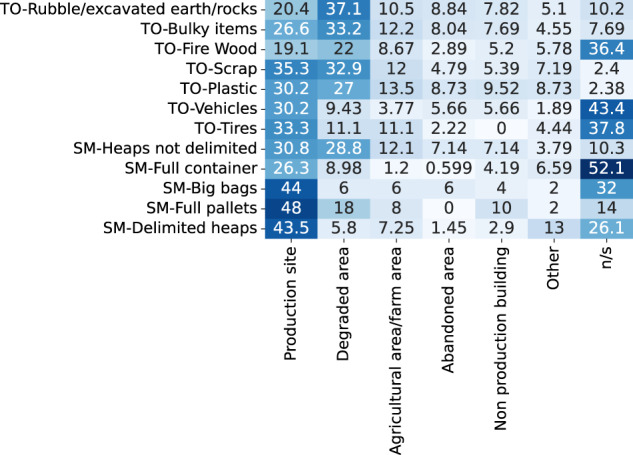
Percentage of TO and SM annotations in different types of areas (“n/s” = not specified).

Figure 8 shows the co-occurrence of the most common TO and SM labels and of the area types. For example, the rubble/excavated earth and rocks class is mostly present in degraded areas.

**Fig. 9:**
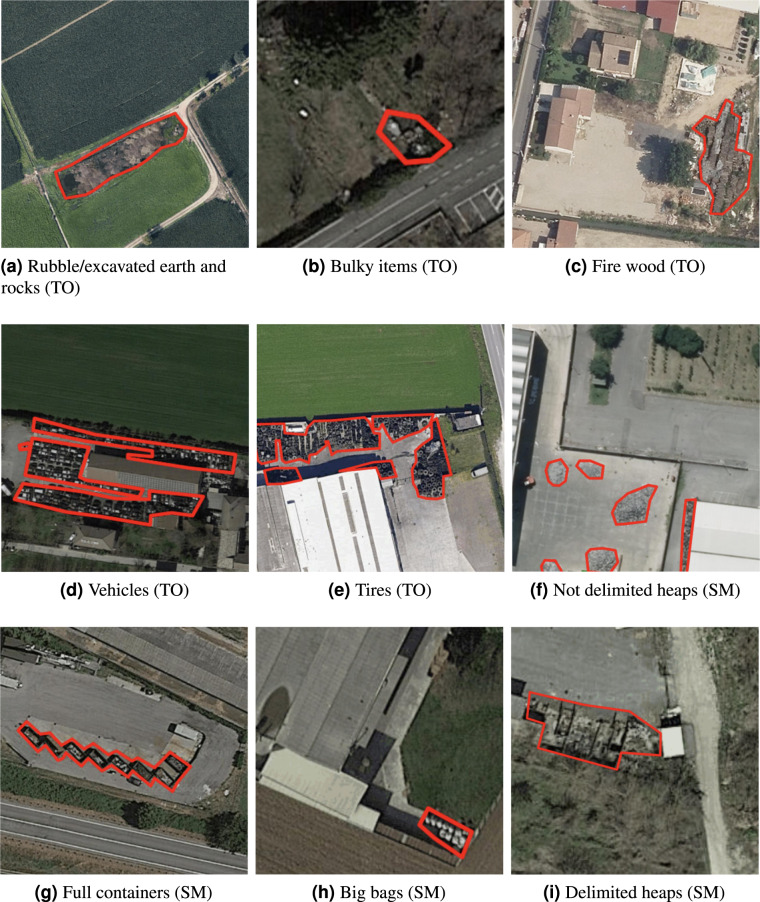
Examples of manually annotated segmentation masks for the various TO and SM classes present in the testing split of the dataset.

For a subset of 169 images belonging to the test set only, the segmentation mask were generated with the ODIN annotation tool^21^ by drawing polygons around the relevant image region. Table 2 lists the 9 categories associated with the segmentation masks and the number of masks per category. Figure 9 shows some examples of the available annotated segmentation masks.

**Table 2:** Type of Object (TO) and Storage Mode (SM) labels and total amount of segmentation masks per label.

Type	Label	#masks
TO	Rubble/excavated earth and rocks	128
TO	Bulky items	69
TO	Fire wood	63
TO	Vehicles	70
TO	Tires	36
SM	Heaps not delimited	172
SM	Containers	188
SM	Big bags	60
SM	Delimited heaps (by barriers/walls/etc)	55
TOTAL		841

## Data Records

The AerialWaste dataset is published in the Zenodo repository^22^ and comprises the following artifacts, which constitute the public part of the dataset:

- Images folder: it contains the images corresponding to each location in the database. The images are not geo-referenced because the coordinates are considered sensitive information.
- Dataset description file: it describes the images in the data set using the JSON format and following the MS COCO guidelines. The description specifies the provenance and metadata of each image.

## Technical Validation

The technical validation of AerialWaste has focused on the adequacy of the dataset for building DL predictive models able to support analysts in the discovery of waste dump sites.

### Training and testing of a waste binary classifier

**Fig. 10:**
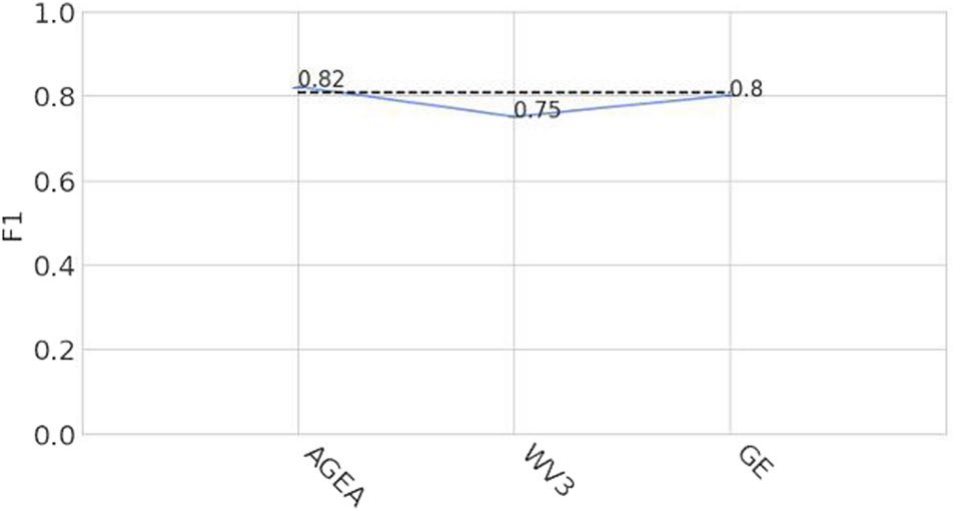
Variation of the F1 score metrics based on the image provenance source.
- We also assessed the impact of image quality by training and testing the same architecture only on the AerialWaste high quality AGEA ortophotos. In this case, the binary classifier achieves 94.5% average precision and 88.2% F1 score. The details of such experiment are reported in^9^.
- The output of the classifier was further validated by experts with the help of Class Activation Maps (CAMs)^24^ to highlight the image regions where the classifier focused its attention.

To cope with the complexity of landfill imagery, in which the recognition of the relevant scenes might need a varying degree of context (e.g., garbage stored in dumpsters vs. scattered in a large area), we trained a multi-scale CNN architecture normally employed in complex scene detection tasks and tested it on a large-scale territory, reporting both qualitative and quantitative evaluation results. The validation procedure can be summarized as follows:

- The dataset is divided in two splits: 75% of the images for training and 25% for testing.
- The train set of the AerialWaste data set was used to train a binary CNN classifier featuring a ResNet50 backbone augmented with Feature Pyramid Network (FPN) links^23^, a technique that improves the detection of items at different scales.
- We evaluated the performance of the architecture on the AerialWaste test set. The binary classifier achieved 87.99% average precision and 80.70% F1 score, with 81.89% precision at 79.54% recall. Figure 10 shows the variation of the classification performances across the different data sources.

#### Classification architecture

**Fig. 11:**
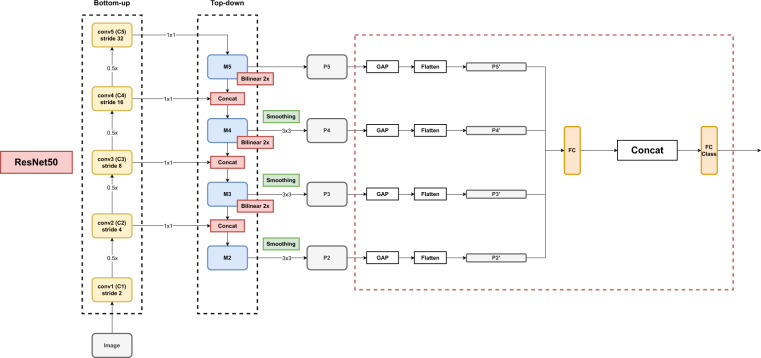
The architecture of the binary classifier extending Resnet50 with FPN links used for the technical validation of the AerialWaste data set.

The binary classifier exploits ResNet50^25^ as the backbone and augments it with a Feature Pyramid Network (FPN) architecture^26^. The FPN improves object detection performances when different scales must be considered^27,28^ and can benefit also classification tasks in which objects of the same class appear with variable sizes. The multi-scale feature pyramid is obtained by combining low resolution semantically strong features with high resolution semantically weaker ones. This is realized by complementing the bottom up feature extraction path typical of CNNs with a top down path that builds the feature pyramid by extracting, adapting, and merging features at multiple levels. Figure 11 shows the structure of the FPN architecture added to the ResNet50 backbone.

The initialization of ResNet50 was performed with transfer learning from ImageNet^29,30^. The best results were obtained by freezing the first two layers during the fine-tuning. In the training phase, data augmentations (flip, rotation, and crop) were applied and the input images were resized to a fixed dimension to cope with different sizes in the same batch and to optimize GPU memory usage. The pixel values were also normalized based on the mean and standard deviation of the data set. After the last FC classification layer, a Sigmoid function is used to obtain a value between 0 and 1, which denotes how confident the model is that the image belongs to the positive class^30,31^. Additional details on how the training was performed are available in the Git repository of the model. A threshold of 0.5 over such a value was used to classify each image.

#### Qualitative assessment and empirical validation with professional photo interpreter

**Fig. 12:**
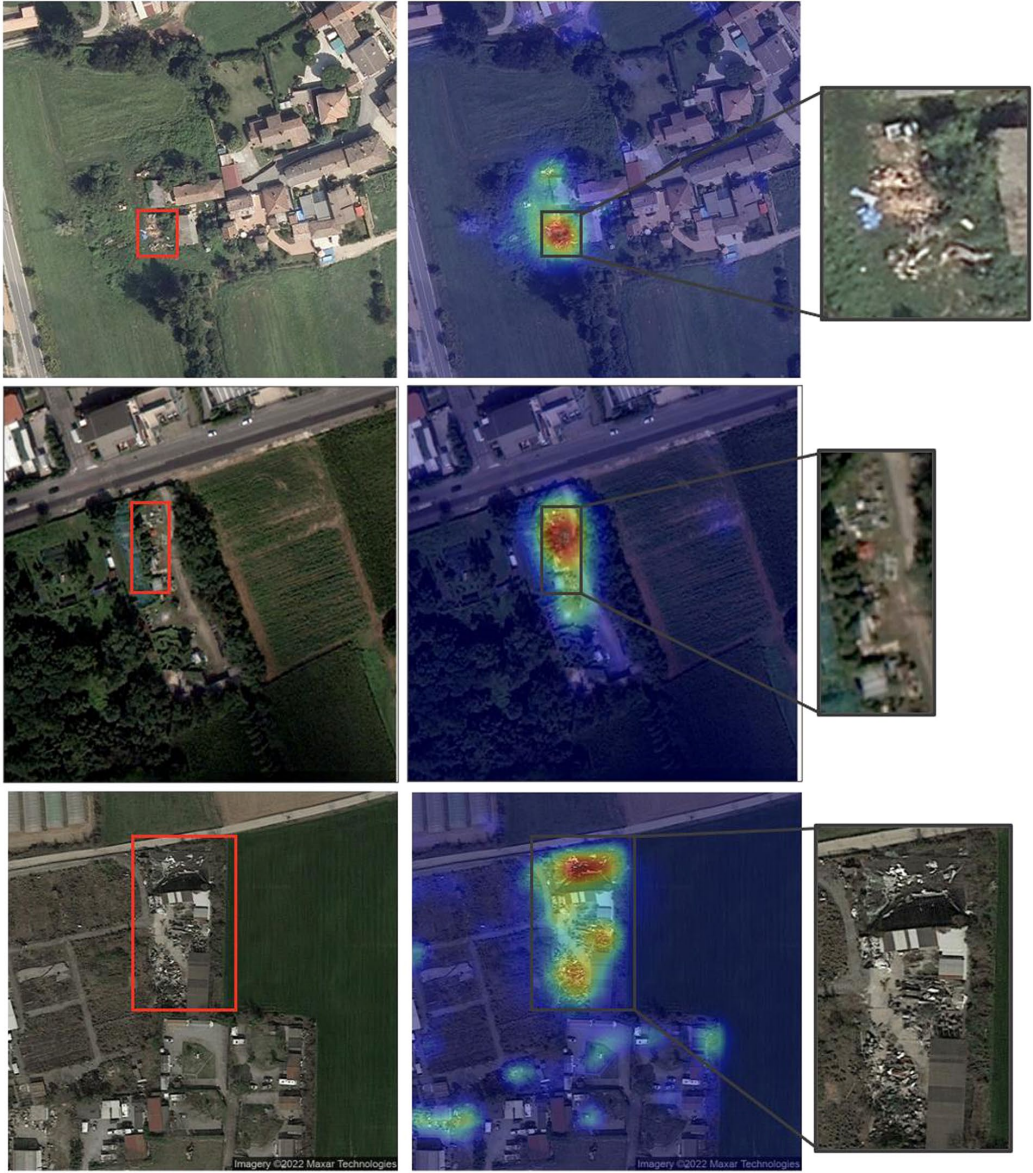
Examples of sites correctly classified as containing waste: AGEA (top), WV3 (center), GE (bottom). In each row, the left image is the input sample overlaid with manually drawn bounding boxes surrounding the areas with waste. The center image shows the heat map derived from the CAM of the positive class. The right image zooms over the bounding box region.

A visual inspection of the results helps understand the quality of the predictions. To understand which objects are responsible for classifying a site as a waste dump, Class Activation Maps (CAMs)^24^ were computed. CAMs are matrices in which each cell contains a value denoting the relevance of the pixel with respect to the class of interest. Figure 12 shows some examples of correctly predicted images overlaid with the heat maps derived from the respective CAMs. The first example illustrates the AGEA image of a residential area in which a backyard contains a small dump of sparse items. The model focuses on the relevant spot, as shown by the CAM heat map. The second example is a WV3 image of a production site with bulky items, well delineated by the CAM heat map. The third example is a GE image of a site disseminated with scrap, plastic, and other unidentified objects, which are correctly highlighted by the heat map peak regions.

The binary classifier trained with the AerialWaste data set was used by ARPA Lombardy analysts to assist in the scanning of the AGEA imagery of a novel territory. A dedicated Inspection Tool was implemented to ease the visualization of the aerial photos, of the model predictions, and of the CAMs. A total of 69 sites were identified as containing waste dumps, of which 65 corresponded to locations predicted by the CNN model as relevant. The model trained only on AGEA orthophotos classified all the 69 locations as relevant. Approximately 50% of the identified sites were located in scenes with a confidence score of the positive class higher than 0.75. The global average confidence score of the sites reported by the analysts is 0.66.

## Usage Notes

### Dataset usage

The dataset can be used to train models for the binary and multi-label classification tasks and for the weakly supervised localization task. The AerialWaste website provides the link to the public repository to download the dataset. Utilities to plot the dataset statistics (e.g., the distribution diagrams presented in this paper) and to visualize it are also provided. A utility class extending the https://pytorch.org/docs/stable/data.html#torch.utils.data.DatasetPyTorch DataSet class is also provided to load the dataset with the PyTorch data processing library.

### Usage with the ODIN diagnostic tool

ODIN^21,32^ is an open source diagnosis framework for generic ML classification tasks and for CV object detection and instance segmentation tasks. ODIN lets developers add custom annotations to their data sets, compute performance metrics split by annotation values, and visualize diagnosis reports. Its annotation functionality was used to draw the segmentation masks illustrated in Fig. 9. The AerialWaste Git repository contains scripts that exploit the ODIN tool for:

- Importing the dataset and obtaining statistics (e.g. samples per class, distribution of annotation values).
- Visualizing the images, with a function to browse the samples and a form-based interface to select them based on a user-defined criterion.
- Visualizing the segmentation masks, with a function to browse the samples annotated with a mask and a form-based interface to select those that meet a user-defined criterion.

Furthermore, when predictions are available, ODIN can be exploited to compute reports of the model performances and to visualize the CAMs and the performance metrics.

### Usage with the inspection tool

The AerialWaste dataset is equipped with an Inspection Tool that supports the visualization of predictions of waste detectors. Its main functions are:

- Map exploration. The user can move around a map of the region of interest by dragging the mouse to different positions or by using an iteration function. The iteration mode superimposes a grid over the area under analysis and lets the user move by changing the grid position with the previous and next commands. The GUI also offers the usual zoom controls.
- Predictions visualization. The user can display the previously computed model output on the map. Predictions are shown by means of color-coded rectangles, where the color indicates the confidence of the model. Very low confidence predictions (below 0.20) are filtered out.
- CAMs Visualization. The user can visualize the CAMs associated with the predictions. This function helps identify the most relevant spots and prevents the oversight of suspicious objects.
- Source selection. The user can choose the map layer among the configured sources. Each source may provide different information. For example, the Google Maps layer provides street names and references to different points of interest, which can be useful to better understand the context.
- Mask definition. The user can mark the presence of waste objects by drawing a polygon around the relevant areas.
- Annotation creation. The user can add or modify metadata about a location. The descriptive fields by default include severity, evidence, environmental risk, waste type, and waste storage mode, plus a free text input for additional information.
- Data export. The user can export the session data in different formats: as a Comma Separated Values (CSV) file to visualize in a spreadsheet or as a Keyhole Markup Language (KML) file to display in a GIS (e.g., QGIS).

**Fig. 13:**
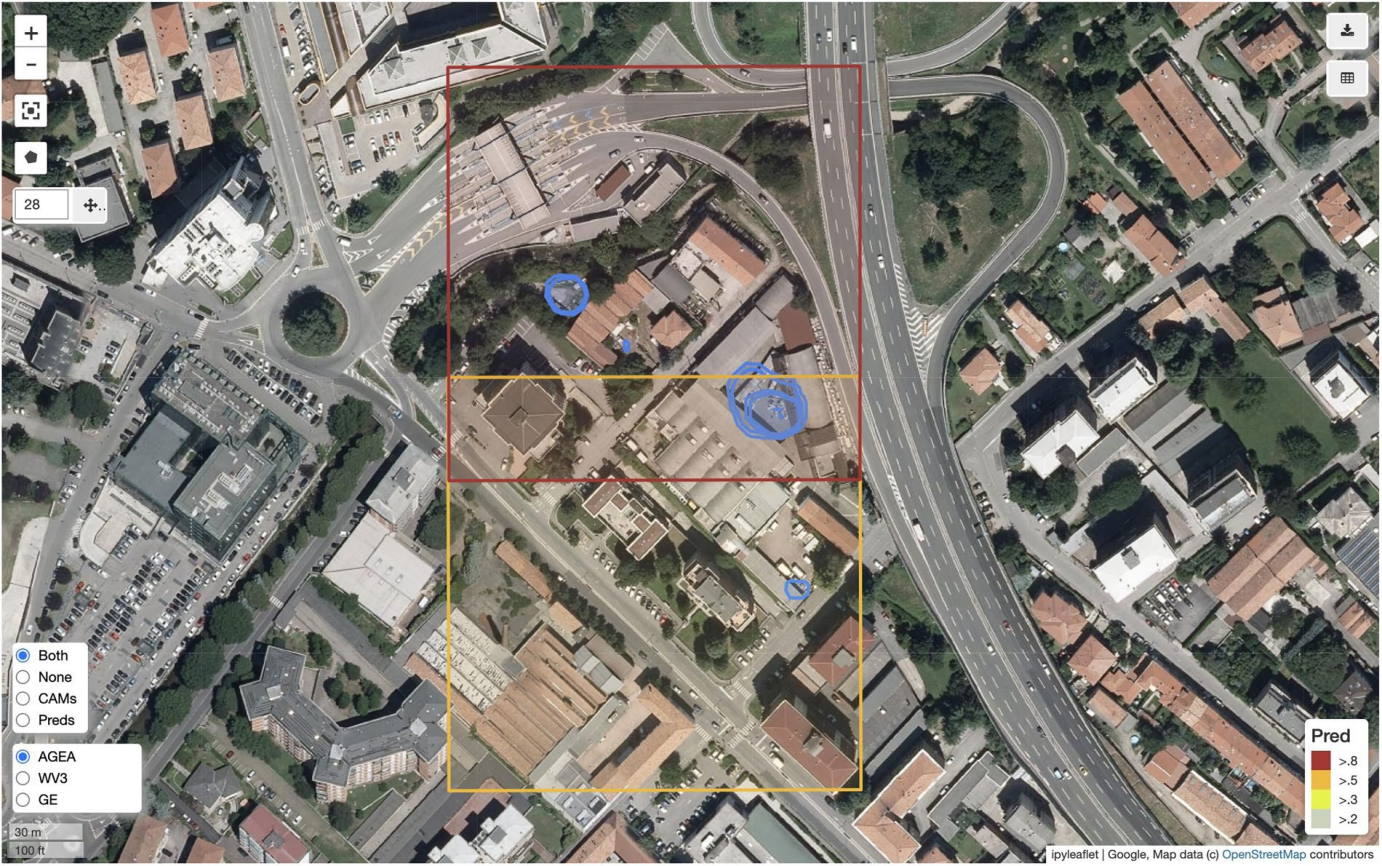
The Inspection Tool showing the model predictions and their confidence levels. The legenda in the lower right corner of the image specifies the color conventions used to display the prediction confidence. The red box corresponds to a scene classified with a score higher than 0.8 while the orange one corresponds to a classification higher than 0.5. In this example, the model was executed using a sliding window of 800 × 800 pixels with an offset of 600 pixels (this is why the prediction areas overlap). The blue polygons display the CAMs generated for the predicted scenes, which highlight the image regions where the model focused the attention.

**Fig. 14:**
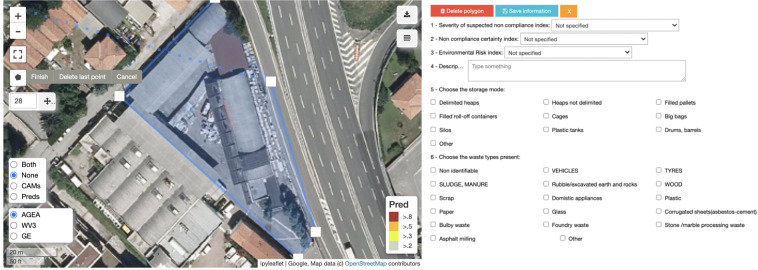
Inspection Tool. Left: the function to create the segmentation mask of a suspicious site. Right: the form-based interface to edit the metadata.

Figure 13 shows the map interface of the Inspection Tool and Fig. 14 illustrates the interface for creating and annotating a site.

### Supported use cases and extension

The AerialWaste dataset is offered to the scientific community for promoting the progress of computer-aided waste discovery in aerial images. It is the first public collection of aerial images portraying landfills and other types of waste dumps curated by professional photo interpreters and made available publicly.

AerialWaste can be used “as is” to train a CV model for the binary (waste/no waste) classification of aerial images. From an application perspective, we found that this level of intelligence is sufficient for environmental agencies seeking to accelerate their territory monitoring processes. When a location is detected as suspicious, the next step is an on-site recognition to ascertain whether the detection is an actual true positive. However, when the area to monitor is large and densely populated the number of positive identifications quickly grows and makes inspecting all the potentially relevant sites impossible. In this case, a multi-class object-based classification is a better aid, because the type of detected waste objects may support the assessment of risk and the prioritization of interventions.

Accurate object localization is the next usage level. In this case, the position and the number of relevant objects can be estimated, which further contributes to proper characterization of the site from remote. The present version of AerialWaste can support the training and testing of weakly supervised localization (WSL) architectures^33,34^ by providing segmentation masks for the images in the test set. Given the high cost of creating object-level masks^34^ we have added to the dataset a number of segmentation masks sufficient to evaluate a WSL model trained with the provided whole-image labels of the object types. We expect that this feature will promote the development of more discriminative predictors advancing the state of the art in computer-aided waste discovery and environmental risk assessment.

The planned extensions of AerialWaste pursue different directions:

- Geographical expansion. The present locations belong to the same Italian region (Lombardy), which albeit very diversified in terms of territory configuration and land use may induce some selection bias. We plan to extend the collaboration to environment agencies in other territories so as to improve the dataset diversity.
- Hard negative sampling. Besides intra-class diversity, aerial images of landfills and of waste disposal sites have also high inter-class similarities. An operational production plant sometimes can be confounded with an abandoned one used for illegal waste storage. We plan to search hard negative examples and add them to the data set.
- Multi-modal imagery. AerialWaste contains only RGB images. The addition of samples acquired with different remote sensing products beyond the visible band could support the design of multi-modal detectors, e.g., exploiting the NIR band to identify the presence of stressed vegetation as a clue for buried waste.
- Multi-temporal imagery. Some sources archive aerial images acquired over time. Analyzing images taken at different dates could provide information on the site activity, e.g., growing or shrinking. Adding time series of images of the same location to AerialWaste may help environmental agencies to plan interventions better, e.g., by prioritizing missions based on the growth rate of the dumping sites.
- Additional metadata. The metadata currently present in AerialWaste are the result of the annotations that experts routinely create in order to characterize the site and are not conceived with the specific purpose of supporting machine learning tasks. We plan to improve the metadata by adhering to the EU waste codes^20^ so as to foster interoperability with future waste discovery datasets and by adding technical annotations supporting the diagnosis of machine learning models.
- Object localization. We are training a WSL component for detecting waste objects with the whole image labels of the data set. We plan to use the segmentation masks output by such a detector as a starting point for the next round of expert annotations. We think that the availability of object mask proposals, albeit not fully accurate, can accelerate the annotation task substantially.

## Data Availability

The data presented in this study, as well as the list of tools employed, are publicly released: • AerialWaste toolkit: Instructions and scripts to visualize it and draw basic statistics are published in the following repository https://github.com/nahitorres/aerialwaste. • AerialWaste model: the models and the code to execute them are released on https://github.com/nahitorres/aerialwaste-model. • Inspection Tool: the Jupyter notebook-based tool is also publicly released on https://github.com/nahitorres/demo-inspection-tool. After the execution of the models in new areas, the predictions can be visualized with the inspection tool. • ODIN: the tool used for the dataset annotation process and for the model evaluation phase is a previous work, available at https://nahitorres.github.io/odin-docs/.
